# A Spatially Correlated Model with Generalized Autoregressive Conditionally Heteroskedastic Structure for Counts of Crimes

**DOI:** 10.3390/e24070892

**Published:** 2022-06-29

**Authors:** Isabel Escudero, José M. Angulo, Jorge Mateu

**Affiliations:** 1 Estadística, Facultad de Ciencias, Escuela Superior Politécnica de Chimborazo, Riobamba EC 060155, Ecuador; aescudero@espoch.edu.es or; 2 Department of Statistics and Operations Research, University of Granada, 18071 Granada, Spain; 3 Department of Mathematics, University Jaume I, 12071 Castellón, Spain; mateu@mat.uji.es

**Keywords:** autoregressive structure, Bayesian inference, B-splines, crimes, MCMC, self-exciting models, spatio-temporal patterns

## Abstract

Crime is a negative phenomenon that affects the daily life of the population and its development. When modeling crime data, assumptions on either the spatial or the temporal relationship between observations are necessary if any statistical analysis is to be performed. In this paper, we structure space–time dependency for count data by considering a stochastic difference equation for the intensity of the space–time process rather than placing structure on a latent space–time process, as Cox processes would do. We introduce a class of spatially correlated self-exciting spatio-temporal models for count data that capture both dependence due to self-excitation, as well as dependence in an underlying spatial process. We follow the principles in Clark and Dixon (2021) but considering a generalized additive structure on spatio-temporal varying covariates. A Bayesian framework is proposed for inference of model parameters. We analyze three distinct crime datasets in the city of Riobamba (Ecuador). Our model fits the data well and provides better predictions than other alternatives.

## 1. Introduction

Modeling time series of counts has received important and growing attention since the 1950s [1,2,3,4,5] and over recent decades (see [6,7,8,9,10]). It is known that some well-known discrete distributions, such as Poisson and negative binomial (NB), can only deal with overdispersion; however, generalized Poisson (GP) and double Poisson (DP) distributions can treat both overdispersion and underdispersion. The latter two models have some shortcomings or limitations. Alternatively, the class of observation-driven models called integer-valued generalized autoregressive conditionally heteroskedastic (INGARCH) models [9,11] shows flexibility in modeling a wide range of overdispersion and underdispersion cases, while possessing properties that make it methodologically appealing and useful in practice.

Although a classical Poisson INGARCH model appears to provide an adequate framework for modeling count time series data and has been applied to several fields, Ref. [12] pointed out that it cannot be employed for modeling negative correlation amongst counts, and it can exclusively include covariates which result in a positive regression term, since otherwise the mean of the Poisson process becomes negative. In addition, the conditional mean is equal to the conditional variance, and this restriction can lead to poor performance of a Poisson INGARCH model with the existence of potential extreme observations.

To overcome these drawbacks, two INGARCH models have been proposed to represent overdispersion or underdispersion in the same framework. These are the DP model [13] and the GP model [14]; see also [15] for a proposal of a Conway–Maxwell (COM) Poisson INGARCH distribution. The reader is referred to the very latest literature in this field [8,9,10].

In the spatial statistics literature, Ref. [16] made an early attempt at structuring spatial relationships for count data by conditionally specifying the data model distribution given a fixed spatial region. This, however, leads to a statistical model that only allows for negative association. Refs. [17,18] demonstrated how the statistical model might be modified to allow for both negative and positive correlation. The important assumption in these models is that the observed count distribution may be conditionally determined from the observed count distribution at spatial neighbors, which is a Markov assumption in space. A good modern review on using spatial structure in econometric models is [19]. While count data in the spatial statistics literature have predominately been addressed through structure in a latent process, in the time series literature it has evolved quite differently. For example, the INGARCH model of [13,20] is a time series model for counts where the data model is Poisson with the expectation that is a function of both previous counts and previous expectations. Ref. [20] demonstrated how the INGARCH(1,1) is analogous to an ARMA(1,1) for counts.

Noting the link between the stationary distribution of the INGARCH(1,1) process and a stochastic process given in [3], often called a self-exciting point process, we have a number of possible point process models [21] that have been shown to be beneficial to representing the dynamics of earthquakes, epidemics, forest fires, traffic accidents, or crimes, which is the motivating problem in this paper. We can find a good number of papers in this latter context, see, as nice examples, [22,23,24,25].

This paper is motivated by the analysis of crime data in the city of Riobamba (Ecuador) provided by three different governmental agencies with the aim of understanding crime behavior and its interaction with society to further help public institutions to enhance proper actions. We note that there are some existing exploratory studies (see [26,27]) that show relevant characteristics of this crime phenomenon. In any case, they do not go further in proposing a spatio-temporal modeling framework.

Following the line of reasoning of [24], we take into account spatial variation by considering a spatial integer-valued generalized autoregressive conditionally heteroskedastic (SPINGARCH) model. This model shares the INGARCH properties while allowing spatial correlation by adding a latent spatially correlated log-Gaussian process [28]. In this framework, and paralleling [24], we formulate a stochastic difference equation for the intensity of the space–time process within a class of spatially correlated self-exciting spatio-temporal models that captures both dependence due to self-excitation, as well as dependence on an underlying spatial process. We indeed consider some extensions from [24] to adapt such methodology to our particular data context. We note that the model in [24] considers a linear regression structure in the covariates which are also constant in time. We structure space–time dependency for our count data through a combination of distance-based covariates that vary naturally in both space and time. We thus consider a B-splines procedure within a generalized additive model that permits it to handle space–time variation and non-linear dependencies. This is indeed another aspect that makes our model different from that of [24]. Our B-splines strategy also allows us to combine covariates that are only varying in space with others (such as the climatological ones) that vary only in time, and with those based on distances that are varying in both space and time. Altogether, our strategy is more flexible and adapts better to the case of our data.

The plan of the paper is the following. Section 2 presents the motivating crime datasets together with the corresponding spatial and temporal covariates. Section 3 introduces the methodological approach and the Bayesian inferential framework. The related computational aspects and corresponding results are described in Section 4. The paper ends with a discussion in Section 5 together with some open lines for future research.

## 2. Description

**Figure 1:**
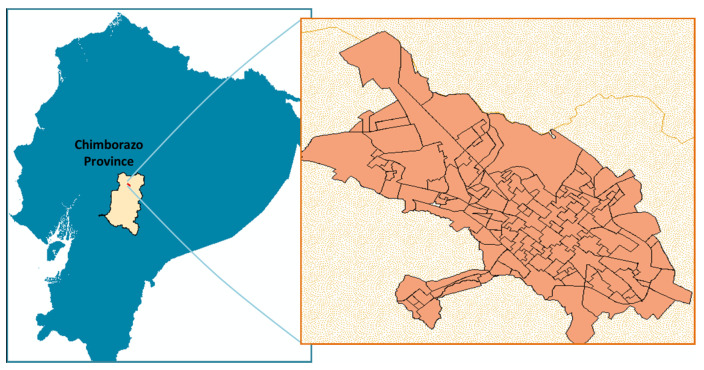
Riobamba within the Chimborazo province in Ecuador.

Citizen insecurity is one of the major problems that affects the development of the population in any country. Riobamba, an Ecuadorian city, is the head of the Riobamba canton and capital of the Chimborazo province (Figure 1). It is located in the inter-Andean region, surrounded by several volcanoes such as Chimborazo, Tungurahua, Altar, and Carihuairazo. Located at 2754 m above sea level, it has a cold Andean climate with an average temperature of 12 °C. According to the 2010 census, this city had 234,170 inhabitants and a population growth of 1.06% until 2014.

Commerce is a typical feature of the city, considered a center of business and employment, and it is the third city with higher education institutes in the country. However, one of the main problems that haunts the place is criminal acts such as assaults, robberies in homes and commercial premises, and intimidation, among others, that cause confusion, concern, and significant general losses to the population [29]. In the Ecuadorian national survey conducted in 2011, the province of Chimborazo ranked seventh with 16.9% of people having been victims of some crime, 73.4% of the population considering that the city is unsafe, and 38.0% having experienced a crime increase in their neighborhood.

According to [30], the Ecuadorian government promoted a set of new policies to reduce crime between 2010 and 2014. These policies involved organized civil society and competent entities. At the end of 2014, the victimization rate, homicide, and robberies decreased but with an increase in societal complaints, a sign of greater confidence in the competent institutions.

**Figure 2:**
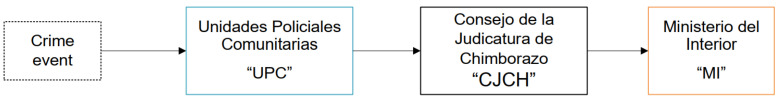
Hierarchical structure for the registration of crimes in Riobamba city.

**Figure 3:**
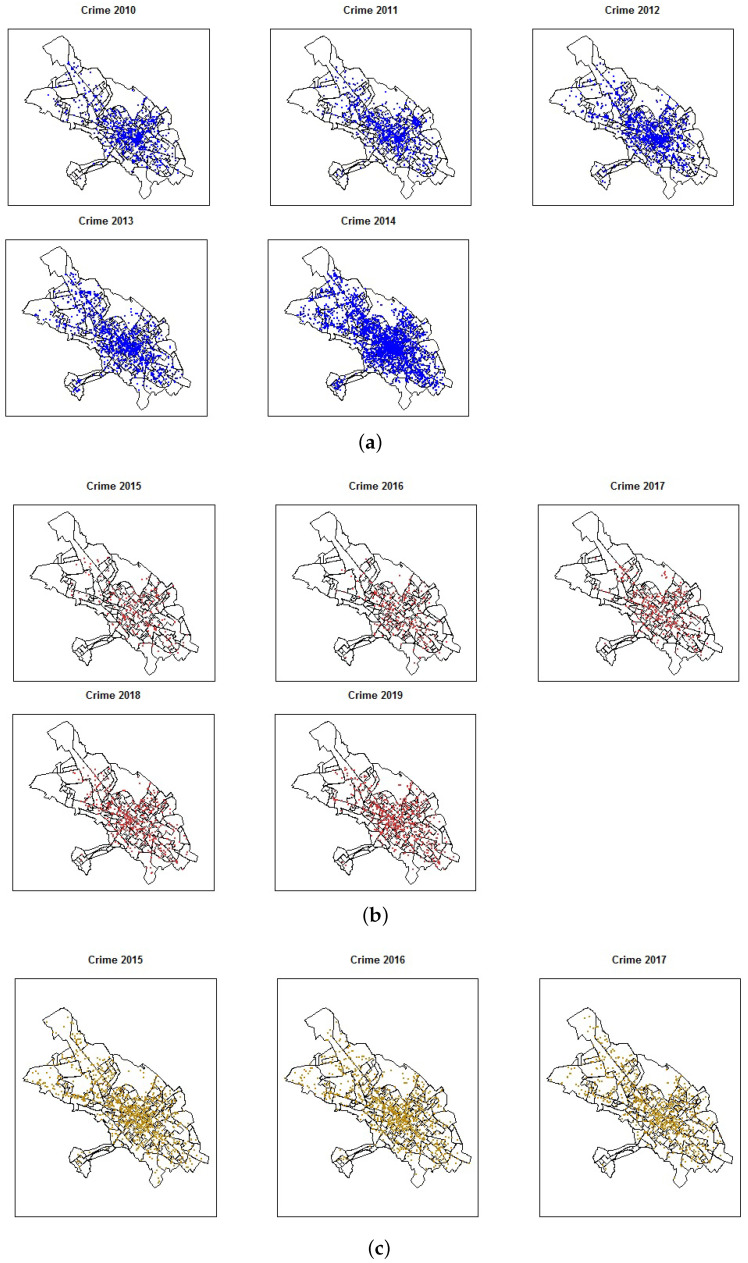
Reported crimes from the three governmental agencies in Riobamba. (**a**) Crimes recorded by MI (2010–2014). (**b**) Flagrant crimes recorded by CJCH (2015–2019). (**c**) Crimes recorded by UPC (2015–2017).

We use data from three governmental agencies whose mission and vision are to guarantee citizen security and social coexistence (Unidades Policiales Comunitarias (UPC), Consejo de la Judicatura de Chimborazo (CJCH), and Ministerio del Interior (MI)). The ideal registration of information dictates that MI saves all reports from the other two institutions, as shown in Figure 2. However, this is far from being true and analyzing the three datasets will prove this anomaly. Figure 3a shows the criminal acts reported from MI for 2010–2014. Figure 3b depicts the flagrant criminal acts recorded by the CJCH for the period 2015–2019, that is, crimes committed with the arrest of the aggressor within 24 h, and finally, Figure 3c shows the crimes registered by the UPC for 2015–2017. The information used here was provided under a confidentiality contract and is not directly available on any website; however, one can consult the criminal data of the Ministerio del Interior from 2015 onwards at http://cifras.ministeriodegobierno.gob.ec/comisioncifras/inicio.php.

**Figure 4:**
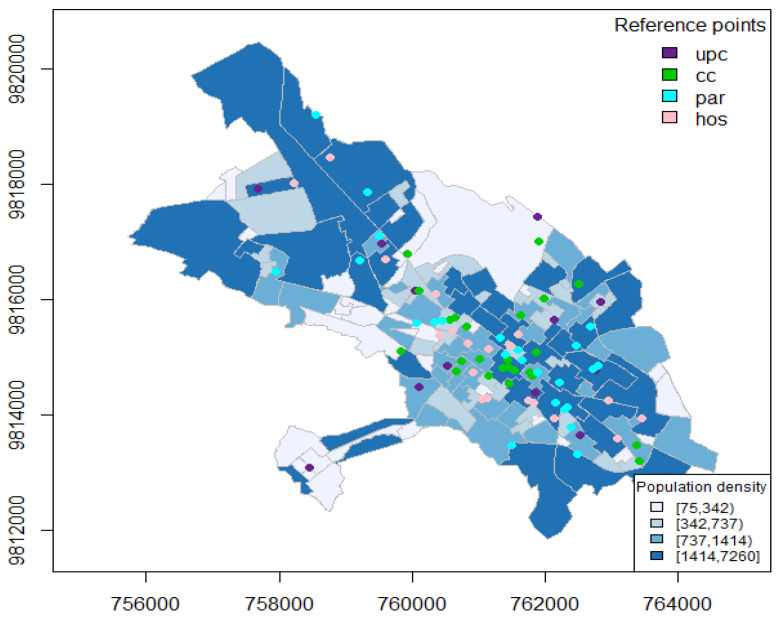
Population density (blue scale), and locations of some landmarks, such as community police units (upc), markets (cc), parks (par), and hospitals (hos).

**Figure 5:**
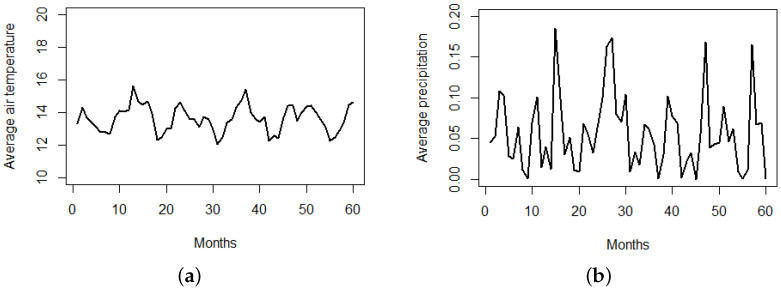
Monthly averages of some climatological variables in Riobamba. (**a**) Temperature. (**b**) Precipitation.

The city of Riobamba is divided into 141 administrative zones as can be seen in Figure 4. The ECU911 (Servicio Integrado de Seguridad) provides the locations in space and time of the crimes, the location of community police units (upc), and the surveillance cameras installed in strategic locations throughout the city (cam). We also consider some important city landmarks describing areas with a higher pedestrian traffic, such as locations of parks (par), hospitals (hos), and markets (cc), that include squares, shopping malls, and supermarkets, and population density at the administrative zone level. In terms of temporal-varying covariates, we consider monthly averages of temperature and precipitation in the city of Riobamba (see Figure 5); these data are available at http://ceaa.espoch.edu.ec:8080/redEma/. These climatological variables are taken into account because there are previous studies (see [25]) that relate them with theft-based crimes.

**Figure 6:**
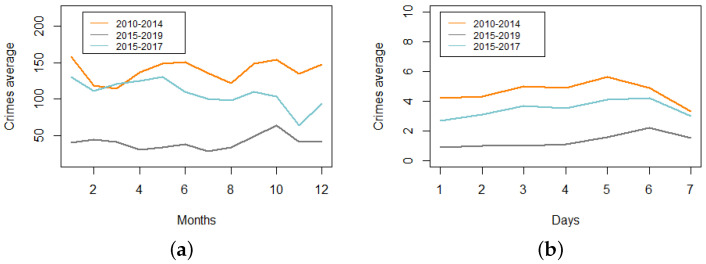
Crime average counts by months (**a**) and days (**b**) for crimes recorded from MI 2010–2014, CJCH 2015–2019, and UPC 2015–2017.

A first exploratory analysis by month highlights that the highest numbers of crimes for general records are found in January, June, and October, flagrant crimes are highest in February, September, and October, and crimes recorded by police increased in January, April, and May (see Figure 6a). This is an indication both that the three types of crime datasets behave differently, and that the month of year plays an important role.

When we look at the data by weekday (see Figure 6b), we find the highest numbers of crimes on Fridays and Saturdays, and according to their spatial location (see Figure 3), there is a high level of crime cases in the downtown area.

**Figure 7:**
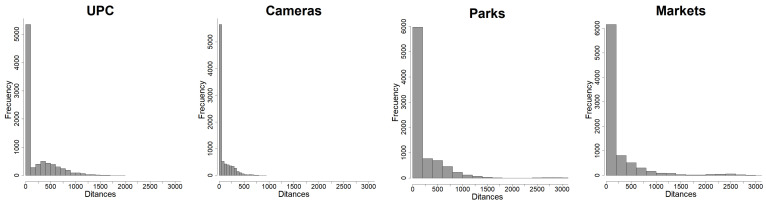
Distance distributions: upc (community police units), cameras (surveillance cameras), parks, and markets (squares, shopping malls, supermarkets).

The locations of the landmarks are taken into account as nearest-neighbor distances between any crime event and the corresponding landmark location. These distances inform about the link between a particular crime and how close one of these landmarks is, and so they inform if landmarks act as attractors or repulsors of crimes. The distributions of these distances are shown in Figure 7, noting how small distances between crime events and landmarks are much more frequent than larger ones, indicating naively that these landmarks could be sources or attractors of crimes.

## 3. Methodology

The overall methodological approach in this paper is to structure space–time dependency for count data through a combination of spatial dependence in a latent process model and temporal dependence in a data model, with exogenous factors that vary over space and/or time. Following [24], we consider a stochastic difference equation for the intensity of the space–time process within a class of spatially correlated self-exciting spatio-temporal models for count data that capture both data model dependence as well as dependence in a latent spatial process. In particular, we focus on a SPINGARCH(1,1) model that overall allows the modeler to define the autocorrelation present in the data and the mean–variance ratio with greater flexibility.

We use the following notation throughout this manuscript. We denote by $\left(s_{1},s_{2},\ldots ,s_{n}\right)$ a vector of spatial (lattice) locations that remain fixed in time, and let *t* be a discrete time period. We denote by $N|s_{i}|$ the spatial neighborhood of lattice location $s_{i}$. Finally, $Y(s_{i},t)$ is the observed process at spatial location $s_{i}$ and time *t*, and $X(s_{i},t)$ is the unobserved latent process. We use a conditional Poisson distribution and place spatio-temporal structure on the covariance of the latent Gaussian process. The data model $Y(s_{i},t)$ can be defined conditionally on the process model $X(s_{i},t)$. As a result, the process model is a function of both observable spatial or temporal covariates and unobservable latent spatial errors.

In our case, the spatio-temporal intensity λ(s,t) provides the process model, and our full model is a stochastic difference equation operating directly on the intensity function. Thus, crime counts in space and time, $Y(s_{i},t)$, are conditionally distributed Poisson random variables for i=1,…,n, i.e., $Y\left(s_{i},t\right)|\lambda \left(s_{i},t\right)∼\mathrm{Pois}\left(\lambda \left(s_{i},t\right)\right)$, with $\lambda (s_{i},t)$ representing the rate at location $s_{i}$ in time *t*. Hence, $E\left[Y\left(s_{i},t\right)|\lambda \left(s_{i},t\right)\right]=\lambda \left(s_{i},t\right)$.

We can assume that a change in crime rate at a specific location and in a specific period is a function of particular geographic features of the location given by $\alpha_{t}={\left(\alpha \left(s_{1},t\right),\alpha \left(s_{2},t\right),\ldots ,\alpha \left(s_{n},t\right)\right)}^{T}$ (also known as reference baseline tension and is simply a function of potentially variable exogenous factors), together with two other factors, a natural deterioration χ, and repeated victimization η.

We propose a SPINGARCH(1,1) model, with $Y(s_{i},t)$ defined conditionally on the intensity $\lambda (s_{i},t)$ which can be modeled using observable spatial and temporal covariates $\alpha (s_{i},t)$, as well as non-observable latent errors $\epsilon_{t}$. Thus, the final model is defined through the following hierarchical structure:

(1) $$Y\left(s_{i},t\right)|\lambda \left(s_{i},t\right)∼\mathrm{Pois}\left(\lambda \left(s_{i},t\right)\right)$$

with

$$\lambda_{t}=\exp\left(X_{t}+\epsilon_{t}\right)+\eta Y_{t-1}+\kappa \lambda_{t-1}$$

$$X_{t}∼\mathrm{Gau}\left(\alpha_{t},{\left(I_{n,n}-\zeta C\right)}^{-1}\sigma^{2}\right)$$

$$\epsilon_{t}∼\mathrm{Gau}\left(0,I_{n,n}\sigma_{\epsilon }^{2}\right),$$

where κ=1−χ represents stress in the absence of repeated victimization, η captures the expected change due to repeated or nearly repeated actions [24], $\lambda_{t}=(\lambda \left(s_{1},t\right),\lambda \left(s_{2},t\right),\ldots ,$$\lambda \left(s_{n},t\right){)}^{T}$ is a Markov chain in ${\left(R^{+}\right)}^{n}$, and the same notation applies for $Y_{t}$ and $X_{t}$. Note that $I_{n,n}$ is the identity matrix, $\sigma^{2}$ is the conditional variance, and ζ controls the amount of spatial dependence in the model not captured by the covariates in $\alpha_{t}$. Large scale spatial structure is accounted for in the latent process $X_{t}$ by the spatial regression parameter $\alpha_{t}$, whereas small scale spatial structure is accounted for by conditionally defining $X_{t}$. For the latter, a conditionally autoregressive (CAR) model is used (through spatially adjacent neighbors):

(2) $$X\left(s_{i},t\right)|X\left(s_{j},t\right),s_{j}\in N\left|s_{i}\right|∼N\left(\mu \left(s_{i},t\right),\sigma^{2}\right)$$

with

$$\mu \left(s_{i},t\right)=\alpha \left(s_{i},t\right)+\zeta \sum_{s_{j}\in N\left|s_{i}\right|}\left[X\left(s_{j},t\right)-\alpha \left(s_{j},t\right)\right].$$

If locations $s_{i}$ and $s_{j}$ are neighbors, the entry (i,j) of *C* will be one. Note that by adding space–time noise $\epsilon \left(s_{i},t\right)\overset{iid}{∼}N\left(0,\sigma_{\epsilon }^{2}\right)$, further variation in the spatio-temporal process is allowed; see some alternative approaches to modeling spatial effects in count data in [19].

### 3.1. Bayesian Inference

The hierarchical model defined above depends on a set of parameters in the final level of the hierarchy given by $\theta =(\eta ,\kappa ,\sigma ,\sigma_{\epsilon },\alpha_{t},\zeta )$, similarly to a classical Besag–York–Mollié (BYM) model [31] which defines a fully Bayesian spatial model (see [32]).

Thus, following [24] we use a Bayesian inferential framework consisting in updating beliefs about θ according to the available data through an a priori density π(θ) and a conditional density or likelihood π(data|θ) to obtain π(θ|data), a posterior density of θ given the data. The a priori joint distribution of the parameters in the model can be expressed as $\pi \left(\theta \right)=\pi \left(\eta |\kappa \right)\pi \left(\kappa \right)\pi \left(\sigma \right)\pi \left(\sigma_{\epsilon }\right)\pi \left(\alpha_{t}\right)\pi \left(\zeta \right)$, where independence is assumed in the background except for η and κ due to the condition η+κ<1. Letting $U\left(s_{i},t\right)=X(s_{i},t)+\epsilon \left(s_{i},t\right)$, the full conditional distribution for θ is given by

(3) $$\pi \left(\theta |Y,U\right)\propto \prod_{t=1}^{T}\pi \left(Y_{t}|\lambda_{t}\right)\pi \left(\lambda_{t}|\lambda_{t-1},Y_{t-1},\theta ,U_{t}\right)\pi \left(U_{t},\theta \right)\pi \left(\lambda_{0}|\theta \right)\pi \left(Y_{0}|\lambda_{0}\right)\pi \left(\theta \right),$$

and for *U* we have

(4) $$\pi \left(U|Y,\theta \right)\propto \prod_{t=1}^{T}\pi \left(Y_{t}|\lambda_{t}\right)\pi \left(\lambda_{t}|\lambda_{t-1},Y_{t-1},\theta ,U_{t}\right)\pi \left(U_{t}|\theta \right)\pi \left(\lambda_{0}|\theta \right)\pi \left(Y_{0}|\lambda_{0}\right).$$

For any inference on the parameters, Markov chain Monte Carlo (MCMC) must take samples of the full latent state density *U*, which requires evaluation of

(5) $$\log\left(U|\alpha_{t},\sigma ,\sigma_{\epsilon },\zeta \right)\propto \frac{-T\times n}{2}\log\left(2\pi \right)+\frac{1}{2}\log\left|\Sigma_{f}^{-1}\left(\theta \right)\right|-\frac{1}{2}{\left(X_{t}-\alpha_{t}\right)}^{T}\Sigma_{f}^{-1}\left(\theta \right)\left(X_{t}-\alpha_{t}\right).$$

Since we can assume that the neighborhood structure is constant for all periods, we can interpret $\Sigma_{f}\left(\theta \right)$ as the full space–time covariance matrix ${\left(I_{T,T}⊗I_{n,n}-C\right)}^{-1}\sigma^{2}+I_{n\times T}\sigma_{\epsilon }^{2}$.

The covariance structure’s sparsity means that the only computations of $\frac{1}{2}{\left(X_{t}-\alpha_{t}\right)}_{t}^{T}\Sigma_{f}^{-1}\left(\theta \right)\left(X_{t}-\alpha_{t}\right)$ that need to occur are for spatial neighbors. Thus, the computation of the determinant $\log|\Sigma_{f}^{-1}\left(\theta \right)|$ is the most challenging element. The specific structure of $\Sigma^{-1}\left(\theta \right)=\left(I_{n,n}-C\right)\left(1/\sigma^{2}\right)$ makes $\log|\Sigma^{-1}\left(\theta \right)|=\frac{n}{2\log\sigma^{2}}+\log\left|I_{n,n}-\zeta N\right|$, where *N* is the neighborhood or adjacency matrix. This in turn can be rewritten as $\log|\Sigma_{f}^{-1}\left(\theta \right)|=\frac{n\times T}{\log\sigma^{2}}+T\log\left|\Sigma^{-1}\left(\theta \right)\right|$, finally resulting in

(6) $$\log|\Sigma_{f}^{-1}\left(\theta \right)|=T\log|\Sigma^{-1}\left(\theta \right)|\propto \frac{n\times T}{\log\sigma^{2}}+T\sum_{j=1}^{n}\left(1-\zeta \chi_{j}\right),$$

with $\chi_{j}$ being the eigenvalues of the neighborhood matrix which depend solely on its structure and not on the parameters.

### 3.2. Generalized Additive Models with B-Splines

The action of the deterministic covariates depending on space or space–time is highly non-linear on the responses. Thus, we have used a generalized additive model (GAM) that supports integrated smoothness estimation addressing the lack of linearity [33]. The GAM results in a more efficient analytical method than the more classical linear models. The relationship between each predictor $x_{i}$ and the mean of the response variable, g(u), is indirect because it is calculated using the smooth (usually splines with polynomial bases [33]) function $f(x_{i})$:

(7) $$g\left(u\right)=\beta_{0}+\sum_{i=1}^{p}f_{i}\left(x_{i}\right).$$

We can also have multivariate versions of the smooth functions per temporal instant. For example,

(8) $$g\left(u\right)=\beta_{0}+f_{t}\left(x_{1},x_{2}\right),$$

with $f_{t}$ being a smooth spatial surface in the *t*-th time. This smooth surface for each *t* can be written as $f_{t}\left(x_{1},x_{2}\right)=B_{s}\psi^{\left(t\right)}$, where $B_{s}=B_{1}⊗B_{2}$ is a B-spline *k*-dimensional basis of dimension $I\times k_{1}k_{2}$ arising from the Kronecker product per row of the marginal B-spline bases for $B_{1}$, $B_{2}$, and $\psi^{\left(t\right)}={\left(\psi_{1}^{\left(t\right)},...,\psi_{k_{1}k_{2}}^{\left(t\right)}\right)}^{T}$. Note that $k_{1}$ and $k_{2}$ are the number of columns of the marginal bases $B_{1}$ and $B_{2}$, respectively, and depend on the number of nodes and degree of polynomials used to generate these bases (see [34]). The generalized cross-validation (GCV) criterion is used to estimate the smoothing parameters, which provide the degree of smoothness. To define the version of smoothing that best fits the data, we test the joint interactions of the spatial covariates with crime.

## 4. Results

The city is divided into m=141 administrative zones (see Figure 4), whose centroids are denoted by $\left\{s_{1},s_{2},\ldots ,s_{141}\right\}$. We compute the adjacency or neighborhood matrix needed in determining the spatial latent process; this is a sparse matrix that permits optimizing the computational costs [35]. Our temporal unit is month, so we consider the number of crimes per zone per month. As the three datasets have different time periods (recall we have for general records 2010–2014, for flagrant crimes 2015–2019, and for police records 2015–2017), we set the time instants (*n*) for the first two cases $t\in \left\{1,2,\ldots ,60\right\}$, and for the latter case $t\in \left\{1,2,\ldots ,36\right\}$.

We refer to Section 2 for a number of covariates considered in our model. In particular, we compute nearest-neighbor distances from each crime to community police units (upc), to surveillance cameras (cam), to markets (cc), to parks (par), and to hospitals (hos). These distances are averaged per administrative zone providing matrices of m×n. The population density (pob) enters the model as a spatial-only covariate of dimension m×1. Although we initially considered two climatological variables (see Figure 5), in an exploratory analysis we noted they were not significant in this particular city, with monthly average temperatures ranging within 12–15 °C and precipitation ranging within 0.00–0.15 mm. Thus, although they are considered in other studies, in our particular region they are not influential on crime.

Although we tested all possible combinations of a multivariate GAM, we found that univariate GAMs provide the best fits. Therefore, we use a univariate generalized additive model with cubic B-splines (denoted by ${\hat{f_{i}}}^{\left[3\right]}$) which allows the incorporation of non-linear relationships between each covariate and the response variable. Our complete GAM model is as follows:

(9) $$\begin{array}{cc} \alpha_{t}= & {\hat{\beta }}_{0}+{\hat{f_{1}}}^{\left[3\right]}\left(upc\right)+{\hat{f_{2}}}^{\left[3\right]}\left(cam\right)+\\ \; & +{\hat{f_{3}}}^{\left[3\right]}\left(cc\right)+{\hat{f_{4}}}^{\left[3\right]}\left(par\right)+{\hat{f_{5}}}^{\left[3\right]}\left(hos\right)+{\hat{f_{6}}}^{\left[3\right]}\left(pob\right). \end{array}$$

In particular, the final significant models for each of the three datasets are the following:

$$\alpha_{t}^{MI}={\hat{\beta }}_{0}+{\hat{f_{2}}}^{\left[3\right]}\left(cam\right)+{\hat{f_{4}}}^{\left[3\right]}\left(par\right),$$

$$\alpha_{t}^{CJCH}={\hat{\beta }}_{0}+{\hat{f_{3}}}^{\left[3\right]}\left(cc\right)+{\hat{f_{6}}}^{\left[3\right]}\left(pob\right),$$

and

$$\alpha_{t}^{UPC}={\hat{\beta }}_{0}+{\hat{f_{1}}}^{\left[3\right]}\left(upc\right)+{\hat{f_{2}}}^{\left[3\right]}\left(cam\right).$$

**Figure 8:**
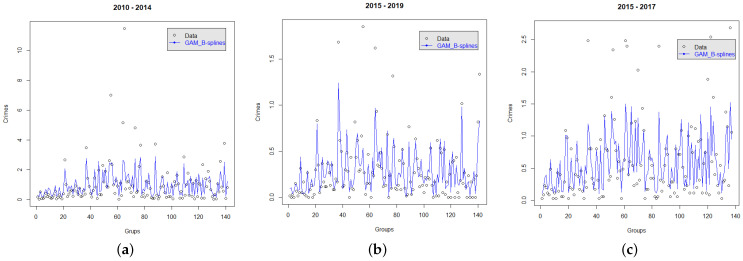
Real crime data and fitted model using B-splines over the exogenous covariates for the three datasets. (**a**) Crimes recorded by the MI. (**b**) Flagrant crimes recorded by the CJCH. (**c**) Crimes recorded by the UPC.

Figure 8 depicts for each dataset the corresponding fitted model with B-splines. We observe how the model fits the real data delineating its behavior well.

**Figure 9:**
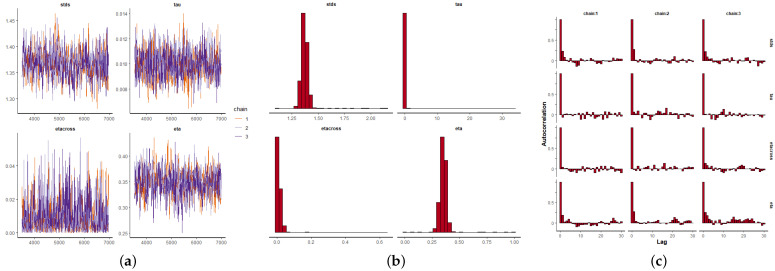
Bayesian inference for the MI data (2010–2014), where: η=eta, κ=etacross, $1/\sigma^{2}=tau$, $\sigma_{\epsilon }=stds$. (**a**) Markov chain convergence. (**b**) Parameter distributions. (**c**) Parameter autocorrelations.

**Figure 10:**
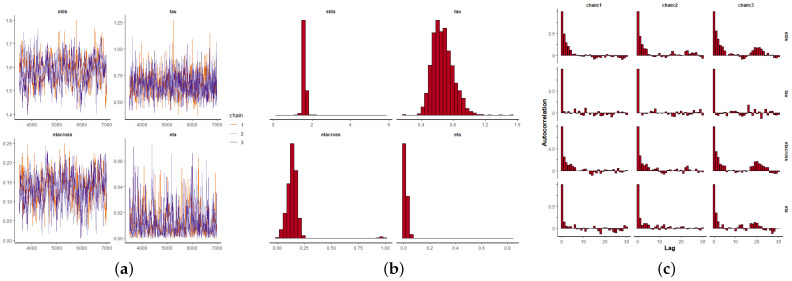
Bayesian inference for the CJCH data (2015–2019), where: η=eta, κ=etacross, $1/\sigma^{2}=tau$, $\sigma_{\epsilon }=stds$. (**a**) Markov chain convergence. (**b**) Parameter distributions. (**c**) Parameter autocorrelations.

**Figure 11:**
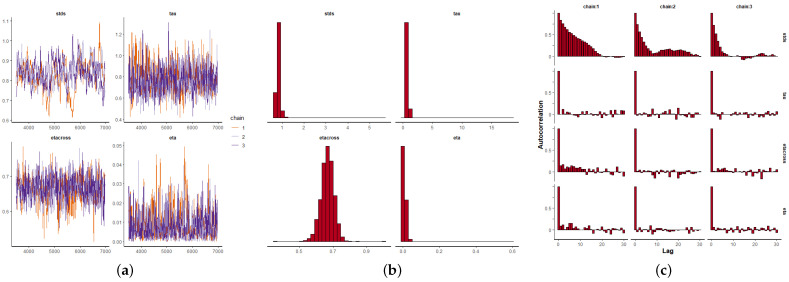
Bayesian inference for the UPC data (2015–2017), where: η=eta, κ=etacross, $1/\sigma^{2}=tau$, $\sigma_{\epsilon }=stds$. (**a**) Markov chain convergence. (**b**) Parameter distributions. (**c**) Parameter autocorrelations.

Once the parameter $\alpha_{t}$ is estimated depending on the covariates, and keeping ζ=0.99 fixed near the edge of the parameter space [24], the remaining parameters $\theta =(\eta ,\kappa ,\sigma ,\sigma_{\epsilon },\alpha_{t})$ are estimated using a Bayesian framework as previously explained. We use informative beta distributions as priors for η and κ, and Cauchy for σ and $\sigma_{\epsilon }$ that minimize the impact on the posterior densities (see also [24]). We run three Markov chains of 70,000 iterations each per parameter, and for each of the three datasets. The first 10,000 iterations are discarded as a burn-in period, and we take samples every 100 iterations to remove any possible sample autocorrelation. Figure 9a, Figure 10a and Figure 11a depict the MCMC chains for the four parameters and for the three datasets. We can see visually the convergence and stability of these chains. The posterior distributions of each of the parameters are shown in Figure 9b, Figure 10b and Figure 11b. We also show the autocorrelation of the parameters as sampled from the posterior distribution, reconfirming the absence of autocorrelation (Figure 9c, Figure 10c and Figure 11c).

Table 1, Table 2 and Table 3 show summary statistics of the estimates and diagnostic statistics for the posterior distributions. Noting that κ (coefficient of deterioration) weights the expected value (i.e., the intensity) while η (coefficient of victimization) weights the data or observations themselves, it is expected that η is larger for MI crimes as from 2015 police interventions were increased in response to an increased number of complaints. In addition, κ is larger for UPC indicating that the model weights the expected intensity more, giving more importance to what is expected than to real data. Recall that the effective sample size (n_eff) and a measure of chain equilibrium (Rhat) are the number of independent draws in the sample and diagnostic statistics on chain convergence, respectively. Rhat values equal or close to 1 are indicative of convergence [36]. For completeness, we also calculate Shannon entropy for each of the parameters associated with each of the three datasets. Taking advantage of the output of the MCMC for each parameter, for which we have a posterior sample of size 1050, we discretize its range length into a number of bins equal to the integer value closest to the square root of the sample size, and calculate the entropy *H* based on these bins. The value of *H* is shown in Table 1, Table 2 and Table 3, and reflects the uncertainty associated with each parameter. A small *H* indicates a small uncertainty in the estimation of the parameter, and thus a larger confidence on its value. Indeed, the estimated parameters with the lowest uncertainty are η and $1/\sigma^{2}$ for the period 2015–2019, κ for 2010–2014, and $\sigma_{\epsilon }^{2}$ for 2015–2017.

**Figure 12:**
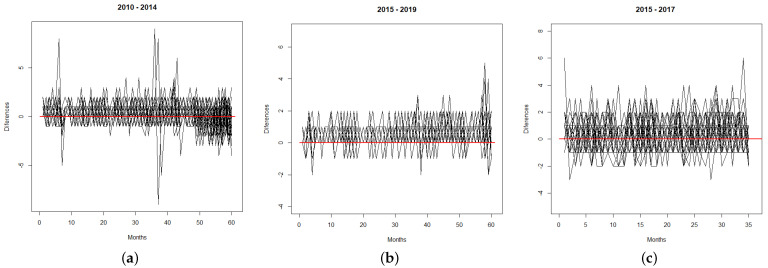
Differences between predicted and real values in space–time. (**a**) Crimes recorded by MI. (**b**) Flagrant crimes recorded by CJCH. (**c**) Crimes recorded by UPC.

As a goodness-of-fit tool, we compute temporal mean square prediction errors (MSPEs) (see Table 4), which report a measure of differences between predicted and real values, noting that the SPINGARCH with cubic B-splines shows the best MSPE values. Additionally, we compute differences between predicted and real values in space–time (see Figure 12), with the corresponding MSPEs being 0.45 (MI), 0.20 (CJCH), and 0.41 (UPC), keeping small in general terms.

**Figure 13:**
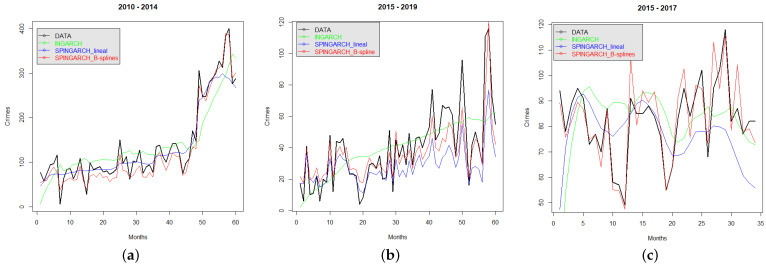
Real and predicted crimes using three competing models: INGARCH(1,1), SPINGARCH(1,1) with exogenous factors being constant in *t*, and SPINGARCH(1,1) with exogenous factors varying on *t* and modeled with cubic B-splines. (**a**) Crimes recorded by MI. (**b**) Flagrant crimes recorded by CJCH. (**c**) Crimes recorded by UPC.

As a final illustration, we compare the temporal prediction of an INGARCH(1,1) model in which there is no spatial effect, of a SPINGARCH(1,1) with exogenous factors entering linearly in a regression fashion, and of our SPINGARCH(1,1) with exogenous factors that vary on space and time and modeled with cubic B-splines. The comparative results are depicted in Figure 13, noting that SPINGARCH(1,1) with smoothed covariates with B-splines provides the best predicted results as they are closer to the real crime data.

For our model, we also report the spatial predictions for the three crime datasets in the city of Riobamba, illustrating that our spatio-temporal model is flexible enough to provide accurate temporal predictions and also spatial predictions.

## 5. Discussion and Conclusions

This manuscript formulates a statistical model that contains both latent spatial and temporal dependencies in the form of a stochastic difference equation for the spatio-temporal intensity. This model is consistent with common beliefs about how violence and crime evolve in space and time. Indeed, the proposed model is a spatially and temporally correlated self-exciting spatio-temporal model that captures both data dependence and dependence on a latent spatial process along the line INGARCH(1,1) models do. Another aspect of our model is that the effect of exogenous covariates is added using non-linear B-splines which improves previous models with only linear forms on the covariates.

We have followed a Bayesian inferential framework as it is flexible and can handle estimation of a large number of parameters with complex structures, such as those considered here in space and time. We are able to estimate neighborhood structure in space and temporal autoregression behavior in time.

**Figure 14:**
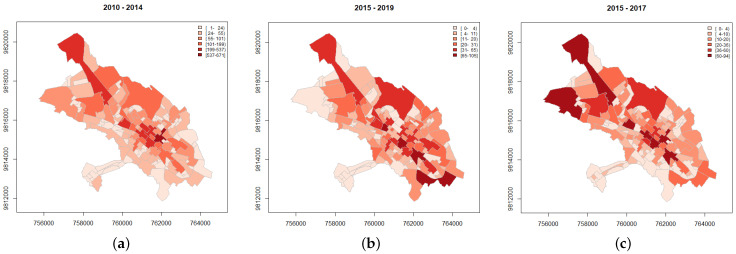
Spatial predictions of crimes from the posterior predictive distributions. (**a**) Crimes recorded by MI. (**b**) Flagrant crimes recorded by CJCH. (**c**) Crimes recorded by UPC.

In analyzing crime data in the city of Riobamba, we were able to detect, by an extensive preliminary search and inspection, which distance-based covariates were most influential and how they entered the prediction model. We highlighted some differences amongst the three types of datasets. For the general registries (dataset for 2010–2014), the minimum distances to surveillance cameras and parks were important because through the monitoring of these cameras, a criminal event was foreseen or taken for granted, and in places such as parks, there is a greater police protection, especially on weekends. For flagrant crimes (2015–2019), the relevant covariates were distances to squares, shopping malls, and supermarkets, and the population density, as having greater population movement contributed to the immediate warning and denunciation of criminal events. Finally, for the police records (2015–2017), distances to cameras and upc had a more representative influence because most of the victims go to the police in the first instance requesting help, regardless of whether the registered criminal event is legally reported or not. The estimation results showed a higher number of crimes in area 65, called San Alfonso, because in the period 2010–2014 the largest market in the city was located there. However, for registered flagrant crimes (2015–2019), we found more predicted cases in zones 37, 55, 76, and 141 (La Dolorosa, La Merced, La Station, and Tubasec, respectively) while for the police files (2015–2017) nine other zones were highlighted (see Figure 14, 2015–2017). These results provide valuable information to governmental entities in charge of citizen security to optimize resources by improving planning, deployment of police units, or patrolling and random verification.

Our data, although in the form of spatio-temporal coordinates, have some limitations. One is that the data provided by governmental entities do not have detailed information on the crime and the characteristics of the events. If they had some additional information, more complex models using mark information could have been used. Another aspect is that INEC (a governmental institution providing and making the data available) has as a minimum unit of study, the parishes, and does not keep statistics by district or by administrative division of the cities. This forced us to randomly disaggregate the data, causing crude approximations of the population.

Open ideas in the context of modeling crime data are many, but identifying crimes happening only in the network of streets in a city enhances the modeling task. In such a case, the Euclidean plane has to be substituted by the network support and this makes things different (see, for example, [37]). We can also think of models for location predictions of the following serial crime using the next hit predictor (NHP) method which adopts the framework of specific self-exciting processes created to characterize the correlations between crimes committed by the same criminal (see [38]).

We finally note that, in [39], the authors studied, by simulation and under different scenarios, the information/complexity transfer from intensity realizations to generated point patterns in spatial log-Gaussian Cox processes. As further research under the model structure proposed in the present paper, we aim at investigating the use of information-complexity measures for assessment of the influential significance of random covariates, involved in the specification of the unobservable latent process, for the response observed patterns. This represents an important challenge due to the intrinsically complex nature of the self-excitation mechanism, and would be particularly useful for comparing different scenarios (type of crimes, urban specificities, etc.), as well as for identification of the relevant explanatory covariates.

## Figures and Tables

**Table 1:** Posterior distribution of the parameters for the MI crimes 2010–2014.

Posterior Parameters	Mean	Sd	2.5%	25.0%	50.0%	75.0%	97.5%	n_eff	Rhat	*H*
η	0.35	0.03	0.29	0.33	0.35	0.37	0.40	685	1.00	2.99
κ	0.01	0.01	0.00	0.00	0.01	0.01	0.04	920	1.00	3.01
$1/\sigma^{2}$	0.01	0.00	0.01	0.01	0.01	0.01	0.01	1028	1.00	2.69
$\sigma_{\epsilon }$	1.37	0.03	1.31	0.35	0.037	1.39	1.42	618	1.01	2.95

**Table 2:** Posterior distribution of the parameters for the CJCH crimes 2015–2019.

Posterior Parameters	Mean	Sd	2.5%	25.0%	50.0%	75.0%	97.5%	n_eff	Rhat	*H*
η	0.01	0.01	0.00	0.00	0.01	0.02	0.50	530	1.00	2.98
κ	0.13	0.04	0.04	0.11	0.14	0.16	0.21	342	1.01	2.86
$1/\sigma^{2}$	0.66	0.12	0.45	0.57	0.65	0.74	0.93	1057	1.00	3.07
$\sigma_{\epsilon }$	1.59	0.06	1.47	1.55	1.59	1.63	1.70	293	1.01	2.68

**Table 3:** Posterior distribution of the parameters for the UPC crimes 2015–2017.

Posterior Parameters	Mean	Sd	2.5%	25.0%	50.0%	75.0%	97.5%	n_eff	Rhat	*H*
η	0.01	0.01	0.00	0.00	0.01	0.01	0.03	635	1.00	2.89
κ	0.67	0.04	0.59	0.64	0.67	0.69	0.74	505	1.00	2.96
$1/\sigma^{2}$	0.77	0.14	0.53	0.67	0.75	0.85	1.08	881	1.00	2.91
$\sigma_{\epsilon }$	0.83	0.07	0.68	0.79	0.83	0.88	0.96	79	1.02	2.70

**Table 4:** Temporal mean square prediction errors (MSPEs).

DATA	INGARCH	SPINGARCH_Lineal	SPINGARCH_B-Splines
MI	1990.97	891.33	364.66
CJCH	351.81	301.39	122.93
UPC	508.88	373.66	56.24

## References

[1] Cox D.R. (1955). Some statistical methods connected with series of events. J. R. Stat. Soc. Ser. B.

[2] Bartlett M.S. (1963). The spectral analysis of point processes. J. R. Stat. Soc. Ser. B.

[3] Hawkes A.G. (1971). Spectra of some self-exciting and mutually exciting point processes. Biometrika.

[4] Hawkes A.G. (1971). Point spectra of some mutually exciting point processes. J. R. Stat. Soc. Ser. B.

[5] Hawkes A.G., Oakes D. (1974). A cluster process representation of a self-exciting process. J. Appl. Probab..

[6] Kedem B., Fokianos K. (2002). Regression Models for Time Series Analysis.

[7] Jung R.C., Tremayne A.R. (2011). Useful models for time series of counts or simply wrong ones?. Adv. Stat. Anal..

[8] Davis R.A., Fokianos K., Holan S.H., Joe H., Livsey J., Lund R., Pipiras V., Ravishanker N. (2021). Count time series: A methodological review. J. Am. Stat. Assoc..

[9] Xu Y., Zhu F. (2022). A new GJR-GARCH model for *Z*-valued time series. J. Time Ser. Anal..

[10] Weiß C.H., Zhu F., Hoshiyar A. (2022). Softplus INGARCH models. Stat. Sin..

[11] Li Q., Chen H., Zhu F. (2021). Robust estimation for Poisson integer-valued GARCH models using a new hybrid loss. J. Syst. Sci. Complex..

[12] Fokianos K., Tjøstheim D. (2011). Log-linear Poisson Autoregression. J. Multivar. Anal..

[13] Heinen A. (2003). Modeling Time Series Count Data: An Autoregressive Conditional Poisson Model.

[14] Zhu F. (2012). Modeling overdispersed or underdispersed count data with generalized Poisson integer-valued GARCH models. J. Math. Anal. Appl..

[15] Zhu F. (2012). Modeling time series of counts with COM-Poisson INGARCH models. Math. Comput. Model..

[16] Besag J. (1974). Spatial interaction and the statistical analysis of lattice systems. J. R. Stat. Soc. Ser. B.

[17] Augustin N.H., McNicol J., Marriott C.A. (2006). Using the truncated auto-Poisson model for spatially correlated counts of vegetation. J. Agric. Biol. Environ. Stat..

[18] Kaiser M.S., Cressie N. (1996). Modeling Poisson variables with positive spatial dependence. Stat. Probab. Lett..

[19] Glaser S. (2017). A Review of Spatial Econometric Models for Count Data.

[20] Ferland R., Latour A., Oraichi D. (2006). Integer-valued GARCH Process. Time Ser. Anal..

[21] Reinhart A. (2018). Rejoinder: A review of self-exciting spatio-temporal point processes and their applications. Stat. Sci..

[22] Mohler G., Short M., Brantingham J., Schoenberg F., Tita G. (2011). Self-exciting point process modeling of crime. J. Am. Stat. Assoc..

[23] Hu T., Zhu X., Duan L., Guo W. (2018). Urban crime prediction based on spatio-temporal Bayesian model. PLoS ONE.

[24] Clark N.J., Dixon P.M. (2021). A class of spatially correlated self-exciting statistical models. Spat. Stat..

[25] Andresen M., Malleson N. (2015). Intra-week spatial-temporal patterns of crime. Crime Sci..

[26] Cepa C., Zabala R., López M. (2018). Proyecto seguridad barrial con involucramiento de los vecinos en Riobamba-Ecuador. Rev. Caribeña Cienc. Soc..

[27] Trejo C., Cisneros J. (2013). La delincuencia en la ciudad de Guayaquil, un análisis espacial de su distribución por delito. Rev. Caribeña Cienc. Soc..

[28] Clark N., Dixon P. (2018). Modeling and estimation for self-exciting spatio-temporal models of terrorist activity. Ann. Appl. Stat..

[29] Chávez Y., Cortez P., Medina P. (2013). Quantification of losses caused by delinquency in Ecuador. Anal. Rev. Anál. Estad..

[30] Castro D., Jácomey J.C., Mancero J. (2015). Seguridad ciudadana en Ecuador: Política ministerial y evaluación de impacto, años 2010–2014. Nova Criminis.

[31] Morris M., Wheeler K., Simpson D., Mooney S., Gelman A., DiMaggio C. (2019). Bayesian hierarchical spatial models: Implementing the Besag York Mollié model in stan. Spat. Spatio-Temporal Epidemiol..

[32] Thamrin S., Alimun (2019). Geographical mapping of dengue fever incidence 2012–2016 in Makassar, Indonesia. IOP Conf. Ser. Earth Environ. Sci..

[33] Taylan P., Weber G.W., Liu L., Yerlikaya-Özkurt F. (2010). On the foundations of parameter estimation for generalized partial linear models with B-splines and continuous optimization. Comput. Math. Appl..

[34] Vicente G., Goicoa T., Ugarte M.D. (2021). Multivariate Bayesian spatio-temporal P-spline models to analyze crimes against women. Biostatistics.

[35] Solarte G., Soto J., Muñoz L. (2013). Matrices dispersas descripción y aplicaciones. Sci. Tech..

[36] Vuong Q.H., La V.P., Nguyen M.H., Ho M.T., Tran T., Ho M.T. (2020). Bayesian analysis for social data: A step-by-step protocol and interpretation. MethodsX.

[37] Gilardi A., Mateu J., Borgoni R., Lovelace R. (2022). Multivariate hierarchical analysis of car crashes data considering a spatial network lattice. J. R. Stat. Soc. Ser. A.

[38] Li Y., Wang T. (2018). Next hit predictor-self-exciting risk modeling for predicting next locations of serial crimes. arXiv.

[39] Medialdea A., Angulo J.M., Mateu J. (2021). Structural complexity and informational transfer in spatial log-Gaussian Cox processes. Entropy.

